# Mesenchymal stem cell-based cell-free strategies: safe and effective treatments for liver injury

**DOI:** 10.1186/s13287-020-01895-1

**Published:** 2020-09-03

**Authors:** Chenxia Hu, Lingfei Zhao, Lingjian Zhang, Qiongling Bao, Lanjuan Li

**Affiliations:** 1 Collaborative Innovation Center for the Diagnosis and Treatment of Infectious Diseases, State Key Laboratory for the Diagnosis and Treatment of Infectious Diseases, |The First Affiliated Hospital, School of Medicine, Zhejiang University, Hangzhou, People’s Republic of China; 2 National Clinical Research Center for Infectious Diseases, The First Affiliated Hospital, School of Medicine, Zhejiang University, Hangzhou, Zhejiang People’s Republic of China; 3 Kidney Disease Center, First Affiliated Hospital, College of Medicine, Zhejiang University, Hangzhou, People’s Republic of China; 4 Key Laboratory of Kidney Disease Prevention and Control Technology, Hangzhou, Zhejiang Province People’s Republic of China; 5 Institute of Nephrology, Zhejiang University, Hangzhou, Zhejiang People’s Republic of China

**Keywords:** Mesenchymal stem cell, Cell-free, Treatment, Liver injury, Cell death

## Abstract

Various hepatoxic factors, such as viruses, drugs, lipid deposition, and autoimmune responses, induce acute or chronic liver injury, and 3.5% of all worldwide deaths result from liver cirrhosis, liver failure, or hepatocellular carcinoma. Liver transplantation is currently limited by few liver donors, expensive surgical costs, and severe immune rejection. Cell therapy, including hepatocyte transplantation and stem cell transplantation, has recently become an attractive option to reduce the overall need for liver transplantation and reduce the wait time for patients. Recent studies showed that mesenchymal stem cell (MSC) administration was a promising therapeutic approach for promoting liver regeneration and repairing liver injury by the migration of cells into liver sites, hepatogenic differentiation, immunoregulation, and paracrine mechanisms. MSCs secrete a large number of molecules into the extracellular space, and soluble proteins, free nucleic acids, lipids, and extracellular vesicles (EVs) effectively repair tissue injury in response to fluctuations in physiological states or pathological conditions. Cell-free-based therapies avoid the potential tumorigenicity, rejection of cells, emboli formation, undesired differentiation, and infection transmission of MSC transplantation. In this review, we focus on the potential mechanisms of MSC-based cell-free strategies for attenuating liver injury in various liver diseases. Secretome-mediated paracrine effects participate in the regulation of the hepatic immune microenvironment and promotion of hepatic epithelial repair. We look forward to completely reversing liver injury through an MSC-based cell-free strategy in regenerative medicine in the near future.

## Introduction

Various hepatoxic factors, such as viruses, drugs, lipid deposition, and autoimmune responses, induce acute or chronic liver injury, [1, 2] and 3.5% of all worldwide deaths result from liver cirrhosis, liver failure, or hepatocellular carcinoma [3]. Orthotopic liver transplantation is performed in end-stage liver disease when the remanent liver tissue is unable to regenerate in sufficient time. However, liver transplantation is currently limited by few liver donors, expensive surgical costs, and severe immune rejection. Cell therapy, including hepatocyte transplantation and stem cell transplantation, has recently become an attractive option to reduce the overall need for liver transplantation and reduce the wait time for patients. However, the clinical application of hepatocyte transplantation is limited by the finite proliferative capacity and missing liver functions of human primary hepatocytes, even though this strategy is simpler, less invasive, and safer than surgery [4]. Stem cells are multipotent cells that are able to proliferate, self-renew, and differentiate into various somatic cells in vitro, and they have attracted much attention for restoring tissue function in vivo with little immune rejection. Stem cells can be classified into three categories: embryonic stem cells (ESCs), induced pluripotent stem cells (iPSCs), and mesenchymal stem cells (MSCs) [5]. ESCs are stem cells derived from early-stage embryos and have high potential to undergo multilineage differentiation. Takahashi and Yamanaka transformed mouse fibroblasts into iPSCs by retroviral transduction of four transcription factors (TFs), Oct4, Sox2, Klf4c, and c-Myc [6]. The multipotency and self-renewal ability of these reprogrammed cells are similar to those of ESCs [7]. MSCs can be isolated from various tissues, such as adipose tissue, bone marrow, the placenta, the amnion, menstrual blood, and other tissues [8–12]. The International Society for Cellular Therapy suggested that MSCs could undergo differentiation into adipocytes, osteocytes, and chondrocytes in vitro [13]. Moreover, these cells have more potency and organ availability compared to those of tissue-specific stem cells, no ethical issues compared to those of ESCs, and less tumorigenicity than iPSCs. Considering the advantages of MSCs, they are widely used to repair tissue injury and in regenerative medicine.

Recent studies indicated that MSC administration was a promising therapeutic approach for promoting liver regeneration and repairing liver injury by cell migration into liver sites, hepatogenic differentiation, immunoregulation, and paracrine mechanisms. Despite their beneficial effects in repairing liver injury, the application of MSCs is limited by aberrant differentiation into undesirable cell types and low engraftment in vivo [14, 15]. Moreover, potential tumorigenicity also inhibits the clinical application of MSCs in current regenerative medicine [16]. MSCs demonstrate potential oncogenic transformation after long-term culture in vitro, and they also have the capacity to support tumor growth in vivo [17, 18]. Hundreds of millions of MSCs are required for transplantation, and the expansion time is approximately 10 weeks before transplantation [19]. MSC transplantation is a risky clinical application since living cells may induce occlusion in the microvasculature [20]. Moreover, transplanted MSCs have a short lifespan and accumulate in lung tissue, which significantly reduces the efficacy of MSC-based regeneration in vivo [21]. However, few MSCs can survive for over 1 week after intravenous administration, which suggests that MSCs participate in repairing tissue injury via paracrine mechanisms [22].

Cell-free-based therapies contribute to liver injury repair via paracrine mechanisms and are safer, cheaper, and more effective treatment options than MSC transplantation. MSCs secrete a large number of molecules into the culture medium, and soluble proteins, free nucleic acids, lipids, and EVs effectively repair tissue injury in response to fluctuations in physiological states or pathological conditions [23]. Soluble and applicable factors can be separated from the MSC-derived secretome and microvesicle fraction via centrifugation, filtration, polymer precipitation-based methodologies, ion exchange chromatography, and size-exclusion chromatography [24]. The manipulation of the MSC-derived secretome is similar to that of pharmaceutical agents, and the storage of the MSC-derived secretome for a long period of time can be performed without toxic cryopreservative agents [21, 25]. Furthermore, the in vitro cell-free MSC-sourced secretome is economical and practical for clinical applications [26]. Cell-free-based therapies avoid the potential tumorigenicity, rejection of cells, emboli formation, undesired differentiation, and infection transmission of MSC transplantation. Although multiple studies have shown significant effects of MSC-derived secretomes in treating liver diseases, the exact key molecules in the MSC secretome need to be further investigated. In this review, we focus on the potential mechanisms by which MSC-based cell-free strategies attenuate liver injury in various liver diseases. Secretome-mediated paracrine effects participate in the regulation of the hepatic immune microenvironment and the promotion of hepatic epithelial repair.

## Current forms of liver injury

The liver consists of multiple functional or interstitial cells, including hepatocytes, cholangiocytes, Kupffer cells, sinusoidal endothelial cells, hepatic stellate cells (HSCs), and dendritic cells, and this organ performs synthesis and secretion functions associated with digestion [27]. However, external injury always initiates a pool of various hepatoxic factors or cytokines in injured sites, which exacerbates internal injury in liver tissue. It is worth noting that the liver may initiate the recovery process and liver regeneration can occur after timely restoration of blood flow or oxygen in vivo via replenishment of cellular adenosine triphosphate (ATP) and controlling excessive reactive oxygen species (ROS); however, ischemia-reperfusion (I/R) injury and the subsequent reintroduction of oxygen-rich blood into liver tissue will promote liver cell apoptosis and exacerbate the progression of liver dysfunction [28]. If long-term ischemia occurs, cell death and cell damage are initiated after the activation of apoptotic or necrotic pathways, and the late stage of liver injury results in the generation of fibrotic tissue and reduced liver function [28]. In addition to hepatic I/R, other chronic liver injury factors, such as viral infection, drugs, high-fat diet consumption, genetic mutations, and biliary tract diseases [29], also largely impair the functions of epithelial cells and activate fibrogenesis and inflammation by activating extracellular matrix (ECM)-producing cells in the liver tissue. These changes further lead to collagen deposition and the generation of irreversible intrahepatic scar tissue in the liver [30]. At the end stage of liver cirrhosis, patients have multiple complications, such as portal hypertension, acute-on-chronic liver failure, or hepatocarcinoma. It is generally accepted that acute liver failure (ALF) occurs in patients without a history of liver disease who develop an international normalized ratio (INR) > 1.5 and any grade of hepatic encephalopathy (HE) within 26 weeks of the onset of illness [31]. On the other hand, acute-on-chronic liver failure (ACLF) is defined as a patient with chronic liver disease who develops liver failure [32]. The balance of hepatocyte cell death and regeneration is disrupted in patients with liver failure, and a majority of hepatocytes undergo apoptosis and necrosis, which are mediated by a multitude of interrelated factors and signals, including caspases, oxidative stress and antioxidants, transcription factors, cytokines, chemokines, and kinases [33].

## Cell death and liver injury

In contrast to most other organs, liver regeneration is also initiated to repair liver injury and compensate for liver function, which is observed in 2/3 partial hepatectomy animal models [34]. On the other hand, hepatocyte injury also results in ATP depletion, which is followed by mitochondrial depolarization, lysosomal breakdown, cell swelling, bleb formation, and cell membrane rupture [35].

Several forms of cell death, including apoptosis, necroptosis, necrosis, and autophagy, are noted in injured liver tissue [36]. However, apoptosis and necrosis are two major forms of cell death in liver diseases. Autophagy is a protective process that delivers cytoplasmic material for degradation in liver tissue to maintain hepatic homeostasis, and hepatic autophagy also includes specific and selective types, including mitophagy and lipophagy [37, 38]. Apoptosis is a kind of programmed cell death that participates in removing damaged cells to protect against external disturbances and maintain liver homeostasis. Hepatocyte necrosis is usually observed in different liver diseases, such as I/R injury or drug-induced liver injury [39]. Cellular morphology undergoes changes, including chromatin condensation, DNA fragmentation, cell shrinkage, and membrane budding, in an ATP-dependent manner after the initiation of hepatocyte apoptosis [40]. Characteristic chromatin condensation and fragmentation result in the packaging of organelles and pyknosis in the cytoplasm, which progresses to apoptosis [41]. In chronic liver disease, the apoptosis rate is higher than the necrosis rate, although both forms of cell death are basic pathogenic mechanisms of liver injury [42]. Mild liver injury factors first promote the translocation of phosphatidylserine from the cytoplasm into the extracellular membrane, subsequently triggering the activation of caspase proteins, the release of mitochondrial factors such as cytochrome c and apoptosis-inducing factor, and DNA fragmentation [40]. Necrosis is characterized by membrane blebbing, cytoplasmic swelling, mitochondrial swelling, mitochondrial calcification, disintegration of organelles, and cell lysis [43]. However, severe liver injury factors promote imbalanced ion homeostasis, DNA digestion, postlytic DNA fragmentation, and cellular necrosis in an energy-independent manner [44]. Necrosis is another kind of cell death that is characterized by uncontrolled damage accompanied by cell membrane damage, cell swelling, and cellular inflammation [45]. Necrosis robustly recruits inflammatory cells into the liver parenchyma and exacerbates the release of inflammatory factors to facilitate liver disease [46].

In conclusion, various liver injury factors such as viruses, drugs, lipid deposition, and autoimmune responses result in different forms of cell death, such as apoptosis or necrosis, depending on the severity of the insult. Different liver injury factors ultimately lead to liver failure when a large number of liver parenchymal cells die and the cell death rate outpaces the liver regenerative capabilities. Moreover, cell death in liver tissue activates the liver inflammatory response and subsequent multiorgan ramifications and carries a grave prognosis.

## The secretion and composition of MSC-derived soluble factors

In vitro cultured MSCs secreted a certain number of soluble factors into Dulbecco’s modified Eagle’s medium (DMEM) to generate MSC-derived conditioned medium (MSC-CM). There are many soluble bioactive factors, including cytokines, chemokines, immunomodulatory molecules, and growth factors, in MSC-CM that exert immunomodulatory functions, inhibit cell death and fibrosis, stimulate vascularization, promote tissue remodeling, and recruit other cells in various tissues. MSC-CM is able to transdifferentiate tissue-specific progenitor cells into somatic cells and participate in tissue injury repair via its paracrine effects [47]. MSC-secreted EVs can encapsulate various paracrine factors, and they can be classified into three categories, exosomes (30–100 nm), microvesicles (100–1000 nm), and apoptotic bodies (500–2000 nm), according to their origin and size [23]. Exosomes are nanosized EVs that originate from the inward budding of the membrane of multivesicular bodies (MVBs) and have remarkable physiological properties. Exosomes are released after the fusion of MVBs with the plasma membrane, and these biological factors are further taken up into target cells [48]. Exosomes can be sedimented by centrifugation at 100,000*g*, and their concentration varies from 1.13 to 1.19 g ml/L in sucrose solutions [49]. The nanometer-sized features made exosomes easy to transfer through blood and other biological fluids. The proteins and miRNAs contained in exosomes are functionally complex and participate in immune regulation, bioenergetics, energy metabolism, antioxidative stress, and tissue regeneration in transplant recipients [50]. Lai et al. demonstrated that the catalytic capacity of exosome-derived enzymes changed according to the surrounding microenvironment. That group concluded that this specificity made exosomes safer than other therapeutic agents [51]. Exosomes protect against the degradation of active ingredients by encapsulating factors within their membranes and facilitating intracellular uptake via endocytosis [52]. In addition, MSC-derived soluble factors deliver multiple factors, such as DNA, mRNA, microRNA, proteins, lipids, and organelles, to recipient tissues and target cells via early endocytosis and subsequent fusion [53, 54]. In particular, a gene ontology analysis showed that various miRNAs, such as miR148a, miR532-5p, miR378, and let-7f, are enriched in the culture medium of MSCs, and these miRNAs can be isolated to regulate different pathways, including cellular transport, angiogenesis, proteolysis, and apoptosis [55–59]. Unique proteins were suggested to exist only in MSC-derived exosomes compared to their parental MSCs [60]. It is worth noting that culture conditions such as hypoxia, ischemia, and inflammation effectively influence the composition of MSC-derived soluble factors [61–63]. On the other hand, soluble factors isolated from MSCs from different sources also have different transcriptome and proteome profiles [64]. MSC-derived soluble factors are preferentially taken up by injured tissues and further mediate cell communication in both adjacent and remote areas via paracrine and endocrine signaling.

## Quality control of EVs

In good manufacturing practice (GMP), there are three important issues for exosome generation: cell culture, purification, and quality control. The main challenge in the GMP of exosomes is quality control since the sources of MSC-producing exosomes are diverse [65]. For further clinical applications and academic studies, appropriate quality control is important when manufacturing clinical-grade EVs with a GMP-compliant process and developing cell-free therapeutics. Determining the quantity, identity, purity, and potency assays of EVs are known as routine quality control criteria.

Total amounts of proteins, lipids, or RNAs can be determined when quantifying EVs; however, nanoparticle tracking analysis (NTA), resistive pulse sensing (RPS), and dynamic light scattering (DLS) are used to determine the number and size of EVs. The most widely used NTA determines the number and size of particles by tracking the Brownian motion of single particles in an aqueous solution [66]. Quantification of EVs remains extremely challenging, and new methods, such as nanoflow cytometry, direct stochastic optical reconstruction microscopy, ExoCounter with optical disc technology, and imaging flow cytometry, have been developed to effectively quantify EVs [67–70]. The purification process can be described as three steps: filtration to remove the cellular debris, concentration of the CM, and exosome isolation from the concentrated CM [71]. To achieve higher specificity, investigators should remove impurities step-by-step. Several proteins, including CD9, CD63, CD81, tumor susceptibility gene (TSG)101, and Alix, which are highly enriched in exosomes, are recommended as specific markers for determining the identity of EVs [72–75]. To determine the purity for quality control, the particle-to-protein, protein-to-lipid, or RNA-to-particle ratios are common markers for monitoring the purity of EVs [72]. There are also some impurities, such as antibiotics and serum, that should be excluded from the cell culture medium when monitoring the removal of potential hazardous factors [76]. On the other hand, another criterion is the absence of several proteins, including histones, lamin A/C, glucose-regulated protein 94, Golgi matrix protein 130, and cytochrome C, since strict cellular localization prohibits the enrichment of these intracellular proteins [72]. Although various biological and biochemical assays are used to determine the potency of EVs or exosomes, it is still difficult to establish a single, standard potency assay since the cargos of EVs in vivo are so complex [77, 78].

## MSC-derived soluble factors are effective for treating liver diseases

**Fig. 1:**
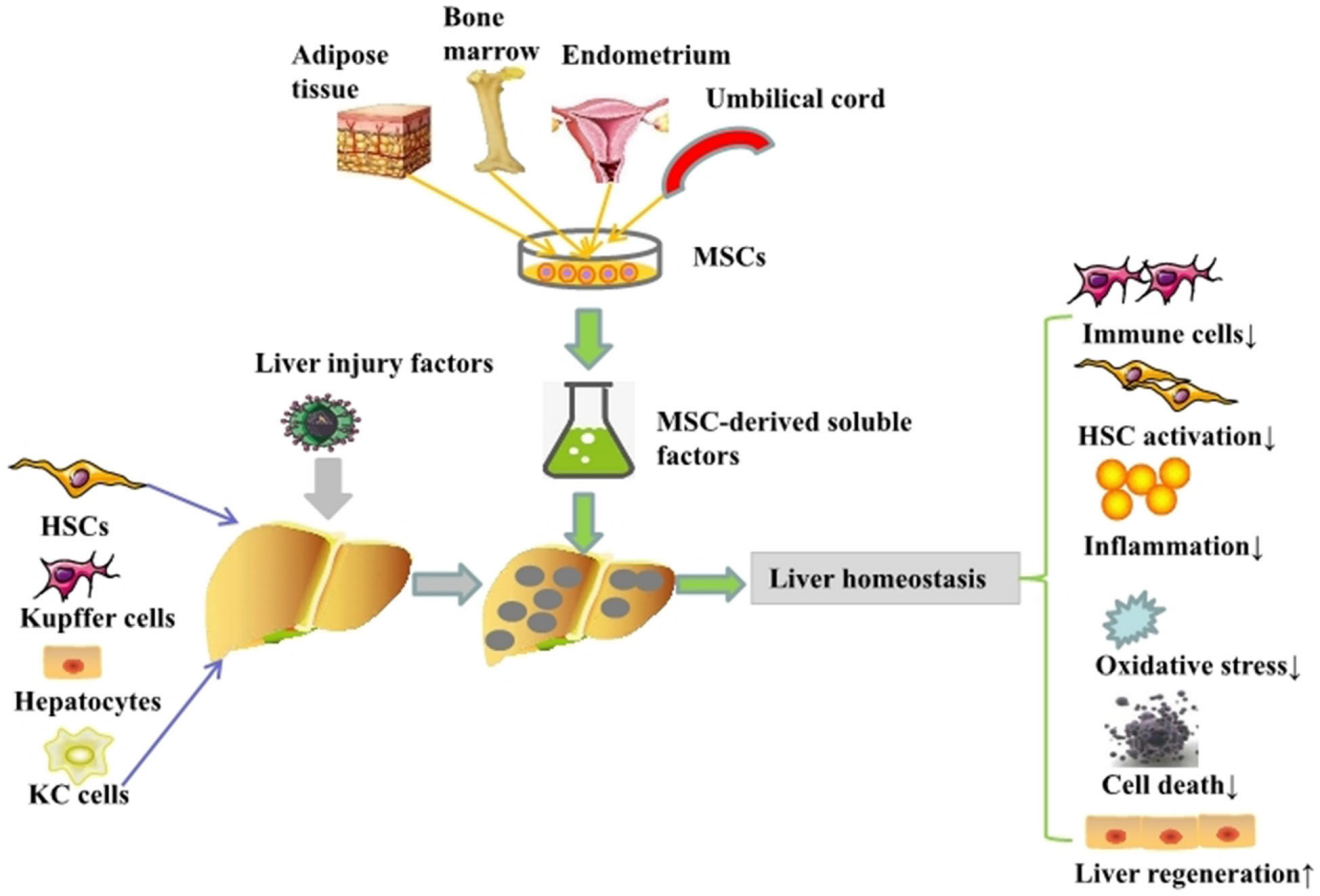
MSCs isolated from various tissues such as adipose tissue, bone marrow, endometrium, umbilical cord, and other tissues are able to secret a large number of soluble factors. These soluble factors have various biological functions including immunoregulation, inhibition of HSC activation, anti-inflammation, and anti-oxidative stress. Moreover, they further decrease the cell death rate and improve liver regeneration for preventing liver injury and maintaining liver homeostasis

Transplantation of MSC-CM and exosomes is the main therapy for treating acute or chronic liver injury (Table 1). Transplantation significantly improved liver function by inhibiting apoptosis, necrosis, oxidative stress, inflammation, infiltration of immune cells, immune rejection, HSC activation, etc. (Fig. 1).

**Table 1:** Transplantation of MSC-CM and exosomes is an effective therapy for attenuating acute or chronic liver injury via different mechanisms

Animal of origin	Tissue of origin	Type of MSC derivatives	Quantity	Route	Liver injury	Model	Effect	Mechanism	Ref.
Rat	Bone marrow	MSC-CM	1 ml (from 1 × 10^6^ MSCs)	Penile vein	Radiation	Rat	Inhibit the apoptosis of SECs; inhibit histopathological changes	Attenuate liver inflammation	[79]
Rat	Bone marrow	25-fold concentrated MSC-CM	1 ml	Penile vein	50% RSLT	Rat	Attenuate liver injury; provide a survival benefit	Reduce apoptosis of hepatocytes and SECs; reduce the secretion of proinflammatory cytokines; reduce the infiltration of neutrophils; activation of Kupffer cells	[80]
Mouse	Bone marrow	25-fold concentrated MSC-CM	200 μl	Tail vein	TAA/CCl_4_	Mouse	Rescue ALF mice; suppress fibrogenesis	Downregulate the number of infiltrating macrophages; convert CD4+ T lymphocytes into anti-inflammatory Tregs and Th2 cells; facilitate HSC death	[81]
Human	Tonsil	20-fold concentrated MSC-CM	200 μl	Tail vein	CCl_4_	Mouse	Reduce liver fibrosis	Attenuate liver inflammation	[82]
Mouse	Bone marrow	Exosomes	10 μg	Intravenous	Con A	Mouse	Decrease the ALT level; decrease the scope of liver necrotic areas; decrease the extent of apoptosis; increase the proliferation of liver cells	Activate anti-inflammation; activate Tregs	[83]
Human	iPSCs	Exosomes	2.5 × 10^12^ particles	Inferior vena cava	Ischemia/reperfusion	Mouse	Decrease the histopathological scores and serum levels of aminotransferases	Inhibit hepatocyte necrosis and sinusoidal congestion; upregulate hepatocyte proliferation	[84]
Human	Umbilical cord	Exosomes	100 μg	Tail vein	D-GalN/LPS	Mouse	Repair damaged liver tissue; decrease serum levels of ALT and AST	Reduce the activity of the NLRP3 inflammasome in macrophages	[85]
Human	Menstrual blood	Exosomes	N/A	Tail vein	D-GalN/LPS	Mouse	Improve liver functions; inhibit hepatocyte apoptosis; increase survival rates	Migrate into liver tissue; reduce the activation of MNCs; decrease the level of the apoptotic protein caspase-3	[86]
Human	Umbilical Cord	Exosomes	6.4 × 10^9^ particles	Tail vein	CCl_4_	Mouse	Decrease the rates of acute liver injury and liver fibrosis	Inhibit oxidative stress; inhibit hepatocyte apoptosis	[87]
Human	Bone marrow	Exosomes	250 mg	Tail vein	CCl_4_	Rat	Alleviate liver fibrosis	Inhibit HSC activation; activate the Wnt/β-catenin pathway	[88]
Human	Umbilical cord	25-fold concentrated MSC-CM	7.2 ml	Liver lobe	CCl_4_	Mouse	Ameliorate liver fibrosis; restore liver functions	Inhibit EMT; protect against hepatocyte apoptosis	[89]
Mice	Adipose tissue	Exosomes	400 μg	Tail vein	D-GalN/LPS	Mouse	Repair damaged liver tissue; decrease serum levels of ALT and AST	Reduce the activation of TXNIP/NLRP3 in macrophages	[90]
Human	Umbilical cord	Exosomes	8 mg/kg, 16 mg/kg, and 32 mg/kg	Tail vein	CCl_4_	Mouse	Rescue ALF; increase survival rates	Deliver GPX1; reduce oxidative stress; reduce apoptosis	[91]

### MSC-CM for treating liver diseases

Infusion of MSC-CM immediately before liver irradiation obviously inhibited the apoptosis of sinusoidal endothelial cells (SECs) and the histopathological changes in the irradiated liver by attenuating liver inflammation [79]. MSC-CM attenuated liver injury and provided a survival benefit in rats with 5% reduced-size liver transplantation (RSLT) by reducing the apoptosis rate of hepatocytes and SECs and reducing the secretion of proinflammatory cytokines, infiltration of neutrophils, and activation of Kupffer cells. Furthermore, the expression of vascular endothelial growth factor (VEGF) and matrix metallopeptidase 9 was also improved to promote liver regeneration in the liver grafts of recipient rats [80]. MSC-derived CM partially ameliorates ALF while significantly suppressing fibrogenesis, necroinflammation, liver apoptosis, and activation of HSCs and improving glycogen synthesis and storage and liver regeneration. Furthermore, MSC-CM reduced infiltrating macrophages, converted CD4^+^ T lymphocytes into anti-inflammatory Tregs and Th2 cells, and facilitated HSC death in liver tissue [81]. Both MSC-CM and interleukin (IL)-1Ra isolated from MSC-CM significantly reduced liver fibrosis by attenuating liver inflammation and inducing alpha-1 type I collagen, transforming growth factor (TGF)-β1, and tissue inhibitor of metalloproteases 1 [82].

### MSC-derived exosomes for treating liver diseases

MSC-derived soluble factors isolated from MSC-CM can also protect against liver inflammation and liver injury. Tamura et al. demonstrated that exosomes escape being trapped in lung tissue and are largely distributed in injured liver tissue. MSC-derived exosomes comparably improved liver functions and hepatocyte proliferation but decreased the apoptosis rate and scope of liver necrotic areas by exerting anti-inflammatory effects and activating Tregs in comparison to those of the MSC group [83]. These exosomes were proven to exert protective effects on liver injury models in a dose-dependent manner. Du et al. demonstrated that MSC-derived exosomes significantly upregulated hepatocyte proliferation in a dose-dependent manner and could directly fuse with target hepatocytes. Consequently, MSC-derived exosomes decreased the histopathological scores and serum levels of aminotransferases by inhibiting hepatocyte necrosis and sinusoidal congestion and upregulating hepatocyte proliferation in the ischemia/reperfusion injury model [84]. Exosomes from human umbilical cord-derived MSCs significantly decreased the serum levels of alanine aminotransferase (ALT) and aspartate aminotransferase (AST) and repaired injured liver tissue in an ALF mouse model after reducing TXNIP/NLRP3 inflammasome activation and its downstream inflammatory factors, including caspase-1, IL-1β, and IL-6 [85]. MSC-derived exosomes dose-dependently inhibited apoptosis in D-galactoside (D-GalN)/lipopolysaccharide (LPS)-treated AML12 cells in vitro. In addition, transplantation of exosomes significantly inhibited hepatocyte apoptosis and increased the survival rates of D-GalN/LPS-induced ALF mice by migrating to liver tissue, inhibiting liver mononuclear cells and downregulating the apoptotic protein caspase-3 [86]. Jiang et al. showed that MSC-derived exosomes alleviated carbon tetrachloride (CCl_4_)-induced acute liver injury and liver fibrosis via the inhibition of oxidative stress and hepatocyte apoptosis and further improved the antioxidant and hepatoprotective effects of bifendate treatment [87]. In addition, exosomes more significantly alleviated CCl_4_-induced liver fibrosis than MSCs, as demonstrated by a reduction in collagen accumulation and enhanced liver functions through a reduction in liver inflammation, upregulation of hepatocyte regeneration, and inhibition of HSC activation through the Wnt/β-catenin pathway [88]. Exosomes from human umbilical cord MSCs significantly protected against liver dysfunction, as shown by decreased ALT levels and TGF-β1 and phosphorylated Smad2 expression. Exosomes also decreased collagen deposition and ameliorated CCl_4_-induced liver fibrosis by inhibiting epithelial-to-mesenchymal transition (EMT), attenuating hepatic inflammation, and eliminating hepatocyte apoptosis [89].

### Exosomes from gene-modified MSCs for treating liver diseases

Intriguingly, gene modification of MSCs provides additional therapeutic effects of exosomes in liver diseases. Liu et al. showed that exosomes from adipose-derived MSCs also improved liver functions through a similar pathway. However, knockout of miR-17 in MSCs abolished the therapeutic effects of exosomes from miR-17-knockout MSCs in ALF mice, which indicated that miR-17 plays a critical role in the anti-inflammatory function of exosomes [90]. A dose-dependent effect was also observed after gavage administration of MSC-derived exosomes. Although 8 mg/kg MSC-derived exosomes failed to rescue ALF animals, 16 and 32 mg/kg MSC-derived exosomes improved the survival rate to 60 and 90%, respectively. In addition, Yan et al. demonstrated that exosomes effectively rescued ALF mice by reducing oxidative stress and apoptosis in liver tissue, and knockdown of glutathione peroxidase 1 (GPX1) in MSCs abrogated the protective effects of exosomes and inhibited the recovery of hepatic oxidative injury in ALF mice [91].

## How can the therapeutic effects of MSC-based cell-free strategies be improved?

To further improve the therapeutic effects, modification of the MSC microenvironments and gene modification of MSCs can consequently alter the composition of proteins and vesicular contents of MSC-derived soluble factors (Table 2).

**Table 2:** Improvement in the efficacy of MSC-derived soluble factors in treating liver diseases via different mechanisms

Animal of origin	Tissue of origin	Pretreatment	Type of MSC derivatives	Quantity	Route	Liver injury	Model	Effect	Mechanism	Ref.
Mouse	Bone marrow	Hypoxia	MSC-CM	5 mg	Intraperitoneal	Acetaminophen	Mouse	Improve biochemical parameters and histological scores	Reduce inflammation; improve liver regeneration	[95]
Human	Adipose	Hypoxia	25-fold concentrated MSC-CM	0.1 ml	Tail vein	Partial hepatectomy	Mouse	Increase the number of proliferative liver cells	Activate JAK/STAT3 signaling	[96]
Mouse	Bone marrow	Hypoxia	Protein composition	10 mg/ml	Intraperitoneal	Acetaminophen	Mouse	Reduce oxygen levels; accelerate healing in damaged liver tissue	Inhibit the activation of inflammation; attenuate hepatocyte necrosis	[97]
Rat	Bone marrow	Coculture with hepatocytes	25-fold concentrated MSC-CM	7.2 ml	Tail vein	D-GalN	Rat	Prolong the survival time of ALF rats	Prevent liver injury; promote liver tissue repair	[98]
Mouse	Bone marrow	miR-223 overexpression	Exosomes	N/A	Intraperitoneal	Liver antigens S100	Mouse	Reverse autoimmune hepatitis	Downregulate the release of cytokines such as NLRP3 and caspase-1	[101]
Mouse	Adipose tissue	miR181-5p overexpression	Exosomes	40 μg	Intrasplenic	CCl_4_	Mouse	Inhibit the deposition of collagen I, vimentin, α-SMA and fibronectin in the liver	Activate autophagy; downregulate Stat3 and Bcl-2 in HSCs	[102]

### Pretreatment or cotreatment for MSC-based cell-free therapy

The common oxygen level is approximately 21% O_2_ for standard cell culture, and this oxygen concentration is not the same as that in the in vivo microenvironment. The physiological oxygen concentration in tissues varies from 1% in cartilage and bone marrow to 12% in peripheral blood [92]. To improve MSC multipotency, proliferation, and secretion of cytoprotective molecules, hypoxic conditions have been proven to significantly improve the survival rate of MSCs in the harsh environments of injury sites after transplantation [93, 94]. CM derived from hypoxic (10% O_2_) MSCs more significantly improved the biochemical parameters and histological results in acetaminophen-induced ALF models by reducing liver inflammation and improving liver regeneration than MSC-CM isolated from a normal oxygen culture environment [95]. CM from MSCs cultured at lower (5%) O_2_ levels more significantly inhibited the activation of inflammation and attenuated hepatocyte necrosis in liver tissue than CM from normoxic MSCs, and hypoxic MSC-derived CM more robustly downregulated oxygen tension and accelerated the healing process in damaged liver tissue of ALF models than CM from normoxic MSCs. Furthermore, CM derived from MSCs cultured in 1% hypoxia showed higher levels of IL-6, tumor necrosis factor (TNF)-α, hepatocyte growth factor (HGF), and VEGF, and these extremely hypoxic CMs significantly increased the number of proliferating cells in liver tissue via the activation of JAK/STAT3 signaling [96]. Proteins isolated from mildly hypoxic MSCs (5% and 10% O_2_) showed better effects in treating acetaminophen-induced ALF than those from normal MSCs by inhibiting inflammatory reactions and reducing hepatocyte necrosis in damaged liver. This group further showed that even moderate hypoxia produced discrete changes in the expression of various subsets of proteins responsible for intracellular respiration and cell signaling [97].

To further improve the therapeutic effects of MSC-CM, MSCs were cocultured with hepatocytes in vitro to improve MSC secretion of protective factors. In comparison to MSC-CM and CM from hepatocytes, the cocultured MSC-CM significantly prolonged the survival time of ALF rats by preventing liver injury and promoting liver tissue repair [98]. Cotreatment with melatonin and MSC-derived exosomes markedly decreased the liver injury score and AST level in the liver I/R group. Intriguingly, the cotreatment group showed comparably low levels of inflammatory factors, apoptosis-related proteins, oxidative stress, DNA damage, and mitochondrial damage to those of the normal group [99].

Culture conditions may influence the regenerative and immunomodulatory capacities of MSC-derived soluble factors, and the optimization of culture conditions will contribute to the further regeneration of liver tissues. According to current studies, pretreatment or cotreatment of MSCs with different factors exerts decisive effects on the therapeutic effects of MSC-based cell-free treatment in recipients. However, the study number is limited, and we suggest exploring the detailed treatments of MSCs and their related mechanisms in depth.

### Soluble factors from MSCs with gene modifications

Valadi et al. first reported that exosomes effectively transferred functional mRNAs or miRNAs into target cells since they found that there were multiple new mouse-derived proteins in human mast cells after the transplantation of mouse MSC-derived exosomes [100]. Current studies have tried to alter the delivered factors to improve the therapeutic effects of MSC-derived soluble factors.

MSCs were infected with pre-miR-223 and transfected with a miR-223 inhibitor to generate exosomes containing miR-223 and exosomes without miR-223, respectively. Exosomes and exosomes^miR-223(+)^ significantly reversed autoimmune hepatitis by downregulating cytokines such as NLRP3 and caspase-1, while exosomes^miR-223(−)^ did not exert protective effects in the experimental model of autoimmune hepatitis. This finding indicated that miR-223 was a key factor participating in the immunoregulation of MSC-derived exosomes to protect against liver injury [101]. In addition, exosomes^miR181-5p(+)^ delivered miR181-5p to injured liver tissue and inhibited the deposition of collagen I, vimentin, α-SMA, and fibronectin in fibrotic livers after activating autophagy and downregulating STAT3 and Bcl-2 in HSCs [102].

## Conclusions

MSC-based cell-free products protect against the potential risks associated with the use of native or engineered MSCs in vivo; moreover, an increasing number of studies have shown that the therapeutic effects of MSC-based cell-free products are equal to those of MSCs. In comparison to MSCs, MSC-derived soluble factors are easier to generate in advance, more stable for storage and transport, and more convenient to control in terms of quality and quantity. As transplantation of MSC-derived soluble factors avoids delivering active cells, infusion of these factors is less immunogenic, less tumorigenic, and less likely to result in exogenic infections, including cytomegalovirus and herpes simplex virus infections [103]. All these advantages make MSC-derived soluble factors promising alternatives to MSC therapy in treating liver diseases. However, current studies have not clarified the detailed factors in MSC-derived soluble factors in vitro and in vivo. The presence of different bioactive molecules mediates the different biological activities of MSC-derived exosomes, which may also influence their final effects in vivo. Furthermore, questions about the characterization, potency, and quantification of MSC-derived exosomes must be addressed before their clinical application is feasible. As MSC-derived soluble factors effectively deliver various cargos in vivo, the identification and purification of these factors should be explored to avoid the administration of potentially harmful vesicles or molecules in the human body. In our opinion, pretreatments or gene modifications may also serve as effective strategies to improve the therapeutic effects of MSC-based cell-free products.

## Data Availability

All data are included in this published article.

## Abbreviations

ESCs: Embryonic stem cells
iPSCs: Induced pluripotent stem cells
MSCs: Mesenchymal stem cells
TFs: Transcription factors
HSCs: Hepatic stellate cells
ATP: Adenosine triphosphate
ROS: Reactive oxygen species
I/R: Ischemia-reperfusion
ECM: Extracellular matrix
ALF: Acute liver failure
INR: International normalized ratio
HE: Hepatic encephalopathy
ACLF: Acute-on-chronic liver failure
DMEM: Dulbecco’s modified Eagle’s medium
MSC-CM: MSC-derived conditioned medium
EVs: Extracellular vesicles
MVBs: Multivesicular bodies
GMP: Good manufacturing practice
NTA: Nanoparticle tracking analysis
RPS: Resistive pulse sensing
DLS: Dynamic light scattering
TSG: Tumor susceptibility gene
SEC: Sinusoidal endothelial cell
RSLT: Reduced-size liver transplantation
VEGF: Vascular endothelial growth factor
IL: Interleukin
TGF: Transforming growth factor
ALT: Alanine aminotransferase
AST: Aspartate aminotransferase
D-GalN: D-Galactoside
LPS: Lipopolysaccharide
CCl_4_: Carbon tetrachloride
EMT: Epithelial-to-mesenchymal transition
GPX1: Glutathione peroxidase1
TNF: Tumor necrosis factor
HGF: Hepatocyte growth factor
